# Coronary Microvascular Angina: A State-of-the-Art Review

**DOI:** 10.3389/fcvm.2022.800918

**Published:** 2022-03-30

**Authors:** Francesco Spione, Victor Arevalos, Rami Gabani, Manel Sabaté, Salvatore Brugaletta

**Affiliations:** ^1^Department of Advanced Biomedical Sciences, University of Naples Federico II, Naples, Italy; ^2^Hospital Clínic, Cardiovascular Clinic Institute, Institut d’Investigacions Biomèdiques August Pi i Sunyer (IDIBAPS), Barcelona, Spain

**Keywords:** coronary flow reserve, microvascular angina, vasospastic angina, coronary microvascular dysfunction, INOCA

## Abstract

Up to 60–70% of patients, undergoing invasive coronary angiography due to angina and demonstrable myocardial ischemia with provocative tests, do not have any obstructive coronary disease. Coronary microvascular angina due to a dysfunction of the coronary microcirculation is the underlying cause in almost 50% of these patients, associated with a bad prognosis and poor quality of life. In recent years, progress has been made in the diagnosis and management of this condition. The aim of this review is to provide an insight into current knowledge of this condition, from current diagnostic methods to the latest treatments.

## Introduction

Angina pectoris is the typical symptom resulting from myocardial ischemia and it affects about 112 million people worldwide (1). Up to 60–70% of patients, undergoing invasive coronary angiography due to angina and demonstrable myocardial ischemia with provocative tests, do not have epicardial coronary disease obstructive enough to explain these symptoms (2, 3). This condition is classified as angina with non-obstructive coronary arteries (ANOCA) and ischemia with non-obstructive coronary arteries (INOCA) when associated with evidence of myocardial ischemia (4). INOCA encompasses the endotypes of epicardial coronary vasospasm and coronary microvascular disfunction (CMD) (5, 6).

The myocardial ischemia consequent to CMD is responsible of what is defined as microvascular angina (MVA). Several studies have demonstrated the prognostic importance of CMD and its association with a worse prognosis (7, 8). Furthermore, this condition is strictly related with a reduced quality of life due to the difficulty in controlling symptoms and the subsequent higher rate of emergency rooms visits, hospital admissions or invasive exams (9).

The aim of this article is to provide an insight into current knowledge of this condition, from current diagnostic methods to the latest treatments.

## Pathophysiology

The coronary vasculature is composed of epicardial arteries (>400 μm), the prearteriolar vessels (400–100 μm), arterioles (<100 μm) and capillaries (<10 μm) (10). The balance between epicardial coronary vessels and the microcirculation is responsible for the myocardial perfusion. The function of prearteriolar vessel is to maintain pressure at the origin of down-stream arterioles within a narrow range when there are changes in coronary perfusion pressure or flow. The more proximal segments are sensitive to changes in flow, whereas the more distal segments to changes in pressure. These vasomotor actions are mainly determined by myogenic mechanisms rather than the direct action of myocardial metabolites (11). The microvasculature compartment (arterioles and capillaries) maintains constant the coronary blood flow (CBF) under a wide range of perfusion pressures by changes in the microcirculation vessel diameter due to myogenic and metabolic mechanisms (12). In this context, the nitric oxide (NO) and other vasodilator substances synthetized by the endothelium play a pivotal role in the modulation of vascular tone and, as consequence, of the myocardial blood flow (13).

**FIGURE 1:**
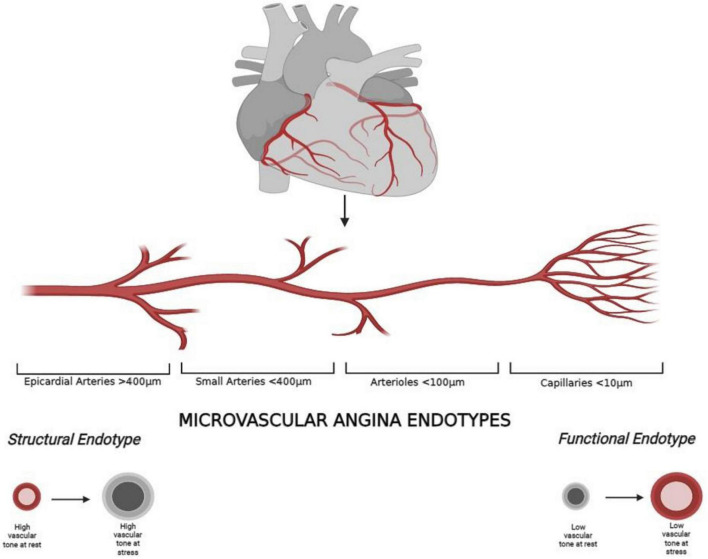
Coronary macro- and microcirculation and microvascular angina endotypes.

CMD has two pathological endotypes: the *structural* endotype and the *functional* endotype (14, 15) (Figure 1). Factors like inflammation or atherosclerosis determine the structural alterations which are associated with a high vascular tone at rest and under vasodilatory stimulus that limit an adequate response. In addition, coronary microembolization is another mechanism addressed as a possible cause, which occurs mainly during interventional manipulation of epicardial plaques. This leads to microinfarction and subsequent inflammatory reaction which may result in alterations in the microcirculation (16). Structural alterations determine a normal rest CBF, a reduction in CBF under stress and in coronary flow reserve (CFR) and higher resistances under stress (4, 15).

On the contrary, the functional endotype is caused by vasomotor disorders that cause an altered response to the vasodilator substances. In particular, in patient with coronary endothelial dysfunction and atherosclerosis, an augmented α-adrenergic coronary vasoconstriction during sympathetic tone activation under stimulus like exercise or mental stress, has been identified as a possible cause (17). The consequence is an exhausted vasodilatory capacity that determines a lower vascular tone at rest and under stress, which is associated with a higher CBF at rest, normal stress CBF, normal resistance under hyperemic stimulus, but reduced CFR (4, 15).

However, the pathophysiology and the underlying mechanisms of CMD are far from being completely understood. Architectural changes, alterations of the NO pathway and increased oxygen demand at rest are considered the most likely responsible for this condition (13).

## Clinical Manifestation

Patients with MVA could have similar manifestations to those with epicardial coronary artery disease, like angina pectoris, atypical angina or angina-equivalent symptoms (18).

Frequently, these patients have effort-induced retrosternal compressive chest pain and/or dyspnea but sometimes symptoms can occur at rest, during the night or when the exercise has finished (5). Noteworthy, there is a gender variation with a higher prevalence of this condition in female, especially postmenopause (19). On the other side, men experience this condition more with atypical symptoms (20).

Furthermore, these patients can have an objective demonstration of myocardial ischemia, obtained with several tests like rest/stress electrocardiography, single photon emission computed tomography (SPECT), positron emission tomography (PET), cardiac magnetic resonance (CMR) or stress echocardiography (5).

## Diagnosis

**FIGURE 2:**
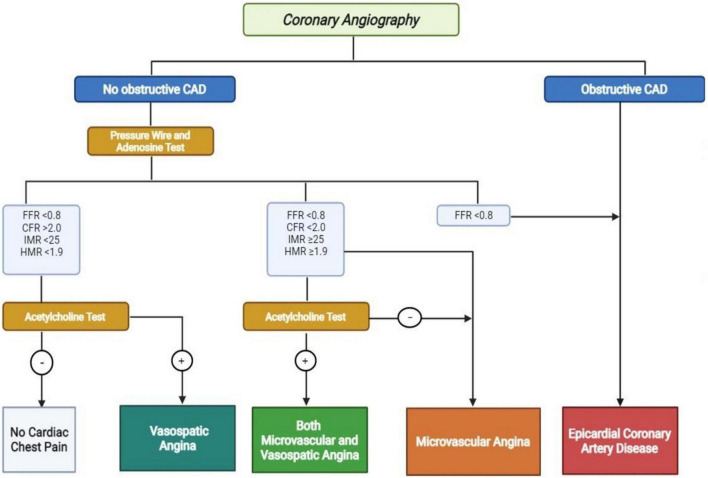
Diagnostic algorithm for no obstructive and obstructive coronary artery disease. CAD, coronary artery disease; FFR, fractional flow reserve; CFR, coronary flow reserve; IMR, index of microvascular resistance; hyperemic microvascular resistance. Positive Acetylcholine Test: >90% reduction in epicardial coronary artery with angina and ECG modifications.

The Coronary Vasomotor Disorder International Study group (COVADIS) provided the diagnostic criteria for MVA (Figure 2) (5):

– Symptoms of myocardial ischemia;
– No-obstructive coronary artery disease (stenosis < 50% or fractional flow reserve (FFR) > 0.80);
– Objective evidence of myocardial ischemia (not mandatory);
– Evidence of impaired coronary microvascular function.

As recommended by 2019 ESC guidelines and by the EAPCI consensus document on INOCA, coronary microvascular function in patients with angina and non-obstructive coronary artery disease should be evaluated invasively (3, 4). The two tools that could be used are an intracoronary Doppler wire or an intracoronary thermodilution-derived method that measure the response of CBF under vasoactive stimuli (21). However, the most used is the thermodilution method that allows to evaluate simultaneously the FFR, CFR and the index of microvascular resistance (IMR) (22). On the other side, the Doppler wire allows the evaluation of hyperemic microvascular resistance (HMR) (23).

The CFR defines the dilator capacity of coronary microcirculation, and it represents the maximal increase of CBF after a vasodilator stimulus. In absence of obstructive coronary artery disease, a value <2.0 identifies the presence of CMD (11). Furthermore, a value IMR ≥ 25 or HMR ≥1.9 identifies the presence of CMD (4).

Adenosine is the drug usually used for this aim at the dose of 140 μg/Kg/min that could be administered intravenously or intracoronary (7). However, the diagnostic accuracy is improved by the use of intracoronary acetylcholine that induces in normal patients a microvascular dilation through the release of NO by endothelial cells (24). The 2019 ESC guidelines recommend its administration in class IIb and it seems that an increase in CBF lower than 50% is correlated with CMD (3).

In the last years, technical innovations have enabled the measurement of absolute coronary flow (AF) and minimal microvascular resistance (MMR) by continuous thermodilution through the use of a dedicated microcatheter and dual-sensor intracoronary guidewires that permit to determine temperature and pressure (24, 25). This microcatheter (Rayflow; Hexacath) has 4 holes at its distal end, where the terminal one is occluded by a guidewire. This guidewire has a temperature sensor linked to a dedicated software (RadiView; Abbott), that allows direct quantitative assessment of microvascular function by measuring coronary flow and microvascular resistances (26). Determination of AF and MMR is potentially the ideal method of measuring coronary flow and resistance, overcoming the limitations of traditional IMR in terms of accuracy, reproducibility and operator independence (24).

### Non-invasive Methods

Although invasive methods represent the gold standard for the diagnosis of CMD, it could be also assessed with non-invasive diagnostic techniques that provide a reliable measure of the CBF (13).

The cardiac PET represents the most validated exam to evaluate the coronary microcirculation. It utilizes positron-emitting radiotracer for the assessment of the myocardial blood flow (MBF), the myocardial perfusion reserve (MPR) and the myocardial flow reserve (MFR). The MPR is the MBF at the maximum stress, whereas the MFR is the ratio of MBF at maximal coronary vasodilation and MBF at rest (27). A value of MFR < 1.5 is related with the presence of CMD (28).

Nevertheless, non-optimal resolution, radiation exposure, high costs, and limited availability represent important limitations of this technique (13).

CMR permits to evaluate the myocardial perfusion and to quantify the blood flow with the use of gadolinium as contrast medium (29). It has the advantage of being radiation-free and to provide a higher spatial resolution. However, the evaluation of MBF with this technique is only performed in research setting (13).

Cardiac computed tomography (CT) can identify the presence of CMD when associated with CT perfusion (CTP) (30). The passage of contrast medium from the coronary circulation to the myocardium, at rest and after adenosine, permits the evaluation of static and dynamic CTP.

The static CTP permits only a semiquantitative and qualitative evaluation of the myocardial perfusion. On the contrary, the dynamic CTP is the equivalent of MBF, which allows to calculate the intramyocardial blood volume. However, its use is still limited in the research setting and it is subject to limitations like radiation exposure and limited availability (13).

Furthermore, also echocardiography can be used to evaluate the coronary microcirculation (21).

Transthoracic Doppler echocardiography evaluates the flow in the middle left anterior descending by color-Doppler and the CBF velocity is assessed by pulsed Doppler wave. This technique is largely available but it is operator-dependent and some patients do not have good echocardiographic window (31, 32).

Finally, myocardial contrast echocardiography is a technique that assesses MBF using the distribution of an echocardiographic contrast medium, but its reliability was only investigated in few studies (33, 34).

## Differential Diagnosis

The other clinical manifestation of INOCA is the vasospastic angina (VSA) which is a vasomotor disorder that determines dynamic epicardial coronary obstruction and, as a consequence, myocardial ischemia (4). The COVADIS defined VSA as a transient total or subtotal occlusion (≥90% constriction) of an epicardial coronary artery which determines a nitrate-responsive angina and ischemic electrocardiographic changes. This situation could occur either spontaneous or in response to a provocative stimulus (6).

A series of triggering stimuli like smoking, drugs, emotional stress, peaks in blood pressure, hyperventilation or allergic reactions can cause VSA due to abnormal function of both vascular smooth muscle and endothelial cells (6).

The diagnosis of VSA is made invasively with a diagnostic guidewire, adenosine test and intracoronary infusion of acetylcholine. Despite normal CFR and microvascular resistance, the acetylcholine test determines ≥ 90% diameter reduction of the epicardial coronary artery with angina and ischemic ECG changes (4, 6).

Sometimes the two conditions of MVA and epicardial VSA can co-exist, with both diagnostic criteria fulfilled during the invasive test. This situation is associated with a worse prognosis (35).

## Management of Microvascular Angina

The MVA pathophysiology is far from being completely understood, regardless of all the progress made so far. This condition is frequently associated with atherosclerosis and endothelial dysfunction due to the fact that traditional cardiovascular risk factors like hypertension, diabetes, smoking, dyslipidemia are present in this class of patients (36, 37). However, the majority of patients remain undertreated due to the lack of evidence-based data about the correct management (38). Nevertheless, Ford et al. showed that a stratified medical therapy combined with a correct invasive diagnostic approach is able to improve symptoms and quality of life in this group of patients (39).

Therefore, it is of utmost importance that the management of this condition is patient-centered and focused on the use of adequate antianginal medications and on the modification of lifestyle and risk factors (4). In particular, the aim of antianginal therapies is to regulate the balance between oxygen supply to the ischemic regions and consumption in the non-ischemic region. This could be achieved by three principal mechanisms: vasodilation that improves coronary or collateral flow; reduction in oxygen consumption in the non-ischemic region; modulation of cardiac metabolism by shifting substrate preference toward glucose and away from fatty acid utilization, given that the former can generate more ATP for any consumed oxygen (40).

The current medical therapy for angina in patients with obstructive coronary artery disease, like beta-blockers and calcium channel blockers, is the standard treatment for MVA.

Medications for the control of blood pressure, like angiotensin-converting enzyme inhibitor, are demonstrated to improve CFR in CMD and can be easily combined with other antianginal drugs like beta-blockers and calcium antagonists (41, 42). Furthermore, due to their anti-inflammatory properties, statins can also be effective by improving CFR (43).

Beta-blockers with vasodilatory properties like nebivolol and carvedilol, showed to provide the most benefits (12). Nebivolol improves CFR thanks to the activation of NO synthesis and inhibition of entothelin-1 (44, 45). On the other hand, carvedilol seems to improve endothelial-dependent microvascular function (46, 47).

Calcium channel blockers like amlodipine, verapamil and diltiazem, induce vascular smooth muscle cell relaxation by blocking the influx of calcium and reduce the myocardial oxygen consumption (48).

Ranolazine may improve exercise capacity and symptoms decreasing calcium and sodium overload in cardiac cells, due to the inhibition of late inward sodium (42). Some studies have shown its role in the improvement of CFR in patients with MVA after its administration, combined with first-line therapy (49, 50).

Other drugs that could give a contribution in the improvement of symptoms in MVA are endothelin receptor antagonists (ERA) and Rho-kinase inhibitors. ERA antagonizes the effects of endothelin by increasing NO availability and thus improving endothelial function (51).

Rho kinase inhibitors inhibit the endothelin pathway by increasing the myosin light chain phosphatase that promotes the unbinding of myosin-acting filaments. This action promotes the vascular smooth muscle cell dilation. However, its role in the treatment of CMD is still under investigation (52, 53).

Nicorandil, ivabradine, and trimetazidine are drugs under investigation to evaluate their potential role in the treatment of CMD. Nicorandil is a mitochondrial ATP-sensitive potassium channel activator that showed some effects in improving symptoms (54). A study demonstrated that ivabradine, which selectively reduces sinus node activity, could improve angina in patients with CMD, but without effects on CFR (55). Finally, trimetazidine limits myocardial ischemia by inhibiting fatty acid oxidation leading to improvements in myocardial glucose utilization (48).

## Prognosis

Patients with CMD have been reported to have a poor prognosis due to several factors like impaired quality of life and higher risk of major cardiovascular adverse events (MACE), especially in the postmenopausal women (56–58). Furthermore, this condition is associated with a higher healthcare cost due to the need of repeat hospitalization and coronary angiograms (59, 60).

The multicenter observational study conducted by Shimokawa et al. showed that MVA was associated with a higher risk of MACE at 1-year follow-up, especially in those patients who had history of hypertension or previous coronary artery disease, without any difference regarding sex or ethnicity (7).

Zhou et al. identified a cut-off value of MFR, derived from non-invasive stress perfusion CMR, which is associated with a worse prognosis. A value ≤ 1.47 was related with a higher incidence of MACE at a median follow-up of 5.5 years and female sex and history of hyperlipemia were independently associated with lower values of MFR (61).

In the iPOWER study by Schroder et al., CMD was assessed by Doppler echocardiography in the left anterior descending artery as coronary flow velocity reserve. A median value of 2.33 was associated with a higher risk of MI and heart failure at a median follow-up of 4.5 years (32).

Gdowski et al. performed a systematic review and meta-analysis of observational studies to determine the association of CMD with outcomes. At a median follow-up from 19 months to 8.5 years, CMD was shown to be associated with a nearly fourfold increase in mortality and a fivefold increase in MACE (62).

Moreover, Toya et al. demonstrated that lower CFR and higher HMR are associated with an increased risk of MACE in a median follow-up of 8 years. Particularly, a per 1-unit increase in CFR predicted MACE with an odds ratio of 0.70 [95% confidence interval (CI): 0.53, 0.92; *p* = 0.01] and a per 1 mmHg/cm/s increase in HMR predicted MACE with an odds ratio of 1.63 (95% CI: 1.20, 2.21; *p* = 0.002) (63).

## Conclusion

MVA is a frequent condition, present in up to 40–50% of patients undergoing coronary angiography for angina and/or positive ischemia tests. Furthermore, it is also associated with a poor cardiovascular prognosis and reduced quality of life, leading to increased pressure on the healthcare system. In recent years, progress has been made in the diagnosis and management of this condition. However, the pathophysiological mechanisms are still not fully understood and the therapeutic management still lacks randomized trials to guide correct treatment. Despite the possibility of using non-invasive diagnostic methods, invasive methods seem to be the most reliable and suitable to make the final diagnosis and to guide the correct therapeutic management. It is of paramount importance that interventional cardiologists adapt to these methods in daily clinical practice in order to guarantee the best possible care for these patients. Further studies are warranted to better understand this condition.

## Author Contributions

FS wrote the first draft of the manuscript. All authors contributed to manuscript revision, read, and approved the submitted version.

## Conflict of Interest

The authors declare that the research was conducted in the absence of any commercial or financial relationships that could be construed as a potential conflict of interest.

## Publisher’s Note

All claims expressed in this article are solely those of the authors and do not necessarily represent those of their affiliated organizations, or those of the publisher, the editors and the reviewers. Any product that may be evaluated in this article, or claim that may be made by its manufacturer, is not guaranteed or endorsed by the publisher.

## Abbreviations

AF: absolute coronary flow
ANOCA: angina with non-obstructive coronary arteries
CBF: coronary blood flow
CFR: coronary flow reserve
CMD: coronary microvascular disfunction
CMR: cardiac magnetic resonance
COVADIS: Coronary Vasomotor Disorder International Study group
CT: Cardiac computed tomography
CTP: perfusion
ERA: endothelin receptor antagonists
FFR: fractional flow reserve
IMR: index of microvascular resistance
INOCA: ischemia with non-obstructive coronary arteries
HMR: hyperemic microvascular resistance
MACE: major cardiovascular adverse events
MBF: myocardial blood flow
MFR: myocardial flow reserve
MMR: minimal microvascular resistance
MPR: myocardial perfusion reserve
MVA: microvascular angina
NO: nitric oxide
PET: positron emission tomography
SPECT: single photon emission computed tomography
VSA: vasospastic angina.
